# Essay on the Endemic Summer Diseases of Floyd County, Indiana

**Published:** 1847-07

**Authors:** H. M. Dowling

**Affiliations:** New Albany, Ia.


### WESTERN JOURNAL

OF

### MEDICINE AND SURGERY.

JULY, 1847.

**Art. I.**—*An Essay on the Endemic Summer Diseases of Floyd County, Indiana.* By H. M. Dowling, M.D., of New Albany, Ia.

No one, I presume, has ever practised medicine in New Albany for a year without meeting with cases of Cholera Infantum. Although more appropriately belonging to the summer months, this disease is seen occasionally as early as May. For the most part, its approaches are gradual. Although liable to become protracted and to assume a chronic character, I have known it to run its course and to terminate fatally in three days. It generally commences with a diarrhea, followed shortly after by more or less nausea and vomiting; very frequently the nausea and vomiting occur simultaneously. In most cases there is considerable febrile disturbance, with fretfulness and an irritable state of the system generally. The tongue at first is coated, but after the disease has continued for some time, it acquires a dry, red, smooth and polished appearance. At first the alvine evacuations are frothy, somewhat consistent, and occasionally tinged with bile, or thin and nearly colorless, and containing small flocculi of mucus. When the disease is completely developed, the discharges rarely exhibit any traces of bilious matter, the secretion of bile being evidently suspended.

These symptoms may all be moderate, and after a day or two, or a few days at most, the child may recover; but very frequently, in spite of all our efforts, we find the disease gaining ground; instead of any amelioration, there is an aggravation of all the symptoms. It is not unusual for the little patient to have twenty, thirty, and even forty passages in twenty-four hours. The exhaustion, which is now extreme, manifests itself in the pale and contracted countenance, the sunken and inanimate eye, the peaked nose, the blanched and shriveled lip, and the deep-colored veins marking the course of the blood as it languidly passes over the hollow temples and the skin-bound forehead. At this time the thirst is very great, and nothing appears so grateful to the little sufferer as cold water, and even this is often rejected as soon as swallowed, so extremely irritable is the stomach. Nourishment of every kind is usually loathed and refused; if received into the stomach and not rejected, such is the enfeebled state of the assimilative powers, that oftentimes it passes undigested rapidly through the bowels. When the disease is about to terminate fatally, aphthæ are apt to appear on the tongue and on the inside of the cheeks. These and a bloated condition of the hands and feet I have always regarded as most unpropitious appearances.

As already stated, the duration of cholera infantum is various; it may terminate fatally in two or three days, or it may run on for weeks or even months before this happens. And as the duration is various, so is the event uncertain. It has been remarked, as the result of frequent and correct observation, that "death sometimes takes place most unexpectedly, and recoveries occur in a state of things apparently hopeless." In protracted cases there is an evident tendency to disease of the brain. I do not at present recollect a case that had a fatal termination, in which I did not consider this result as the sequel of some direct cerebral affection.

Believing that the pathology of cholera infantum consists mainly in torpor of the hepatic and cutaneous functions, in connection with cerebral irritation from dentition, and that these exert a powerful influence in developing and sustaining that irritable condition of the alimentary canal upon which the characteristic phenomena of the disease depend, I have little or no confidence in any remedies that will not restore the one, and at the same time control and obviate the other. I therefore generally begin the treatment by lancing the child's gums if these be either red or tumid, and give powders composed of calomel and ipecac., calomel and rhubarb, or calomel and Dover's powder, and repeat the doses at longer or shorter intervals according to circumstances. If there be pain or tormina in the bowels, I direct the application of a warm poultice in which a little flour of mustard has been incorporated. I am also in the habit of putting small blisters behind the ears, as sources of counter-irritation, and believe they act a very important part, not only in withdrawing disease from the head when present, but in warding off an attack when threatened.

In no disease is a critical inspection of the evacuations so important as in this. By it we learn to a great extent not only the amount of disease, but also the effect of our remedies. And in proportion as the discharges assume a bilious appearance, just in the same proportion are our medicines acting beneficially, and, as a matter of course, the disease putting on a more encouraging aspect. With the restoration of a healthy hepatic secretion we generally find a clean tongue, a moist skin and some appetite. Under these favorable circumstances, provided due regard be paid to regimen, the patient will soon recover. But if a diarrhea supervene, or the liver appear to be too active, I have found it advisable to put a blister to the epigastrium, and to give at the same time Dover's powder, or, what is sometimes much better, a few drops of tincture of opium. Occasionally, and when cerebral symptoms do not contraindicate it, I employ astringents and absorbents in combination with opiates. The common chalk mixture I frequently use, and esteem it the best of its class. The acetate of lead in union with the compound powder of ipecac., I have found an excellent remedy, especially when the fever is moderate and the evacuations are watery and frequent. The vegetable astringents have so often disappointed me that I now seldom prescribe them. I would make an exception here, however, in favor of the blackberry root; this I have frequently seen employed with the happiest results. It would seem to be more especially applicable in the advanced stages of the disease, and does good apparently by arresting the secretions and imparting tone to the mucous lining of the alimentary canal. The spirits of turpentine, so highly commended by Dr. Condie, I have seldom used in this affection; the instances in which I have seen it tried did not impress me very favorably as to its virtues.

In this truly severe and often most troublesome disease, the physician is obliged to vary his prescriptions very frequently. Indeed, such a heavy contribution, generally, is imposed on his resources, that he is often at his wit's end to find a new and appropriate remedy. One thing we are sure to discover in the management of cholera infantum, and that is, that our remedies will do no good unless the strictest attention be paid to our dietetic injunctions. I have more than once lost patients in this disease, when I conscientiously believed a different result would have taken place had not a false tenderness on the part of the mother induced her to administer improper articles of food. Considering the proper regulation of diet a vital part of the treatment of cholera infantum, without which few patients can recover, I would say on this head most emphatically, that no nourishment should ever be allowed except it be of the blandest quality. The parent's milk, being the most natural, is also the most proper, as a general rule. But I have sometimes derived benefit in withholding even this for a time, and substituting ice-water, or very thin arrow-root or tapioca. In the highly irritable state of the stomach often met with in this disease, the greatest error, I conceive, is committed in attempting to give nourishment. It is decidedly better to give nothing at all, or only a few drops of ice-water until the stomach is able to receive nourishment.

The next disease to be noticed is *Cholera Morbus:*

Although by no means so common as cholera infantum, it is yet frequent enough to merit special attention. I regard this as one of the most formidable diseases we are called to combat. Having its seat in the alimentary canal, it is characterized by frequent and violent vomiting and purging, with severe tormina and cramps in the muscles of the abdominal parietes and extremities. Its invasion is generally sudden, and so rapid are the symptoms, that, unless relief be obtained, the patient is soon reduced to a most alarming state of exhaustion. The alvine evacuations are generally very copious and watery, and not unfrequently of the genuine rice-water character. The thirst is excessive, but everything taken into the stomach is almost instantaneously rejected. The pulse is small, weak, intermitting, and sometimes not to be felt at all. The extremities are cold, and, with the forehead, frequently bathed in perspiration. The countenance is altered, and is generally expressive of the most intense anguish. This disease, however, notwithstanding its extreme violence and rapidity, is rarely fatal—not that it cures itself, but being so violent in the commencement, and so universally dreaded, there is seldom any delay in procuring medical aid. I do not recollect having seen or heard of a fatal case since I came to New Albany. Although this disease is often attributed to the eating of indigestible articles of food, such as unripe fruits, fresh pork and the like, yet it is obvious that these act only as temporary and exciting causes.

The pathology of cholera morbus, like that of cholera infantum, consists in engorgement and consequent torpor of the liver, and indeed of the whole of what is denominated the portal system. Never was there a more false or bungling appellation than that by which this disease is generally known. Cholera morbus signifies literally a bile-flowing disease. Now, so far from the name being expressive of what the malady really is, I venture to say that in ninety-nine cases of a hundred it is the very reverse. I have never known an instance where the least trace of bile was present in the evacuations at the outset of the disease. When bile appears, it is hailed as the harbinger of hope.

Called to a case of this disease, our first care should be to relieve the vomiting and cramps. To accomplish this, I am in the habit of applying sinapisms immediately to the epigastrium and to the extremities. I then give equal quantities (say half a drachm each) of laudanum and lavender compound. If this be rejected, I repeat the dose. I allow the patient no kind of drink. If ice can be had, I permit him to take a piece in his mouth and to swallow it as it dissolves. This is exceedingly grateful to the patient, and appears to satisfy his thirst. Nearly every systematic writer recommends the free use of diluents in this disease. I have for a long time considered this an error, and hence am particular in prohibiting every species of drink so long as irritability of the stomach continues. After a respite has been obtained by the means already specified, I begin with powders composed of calomel and opium, (ten grains of the former and one grain of the latter,) and repeat the dose every two, three or four hours, according to circumstances. If the exhaustion be very great, I allow the patient a little warm brandy, and so soon as the stomach will receive it, some well prepared chicken soup, as hot as he can sup it, beginning with a very small quantity. In a few hours the patient is out of danger. If, however, there should come on reaction, with pain in the hypogastrium, it will be necessary to administer cathartics and to apply a blister. In a word, the subsequent treatment resolves itself substantially into that proper for bilious fever.

Another of our hot-weather diseases is *Dysentery:*

Although severe and dangerous, this, fortunately, is not of very frequent occurrence. I have met with it, I think, much oftener in children than in adults. It appears to affect the larger bowels principally, and is characterized by frequent mucous and bloody stools, tormina and tenesmus, with more or less accompanying fever. There is often, also, considerable pain and difficulty in voiding urine. In very bad cases the patient's strength is rapidly exhausted. The pulse sinks and becomes exceedingly small and frequent, and the countenance assumes the true Hippocratic cast. The tongue for the most part is furred, but in inveterate cases it is found clean, smooth and red. From the commencement and throughout the whole course, so long as the disease holds sway, little or no fæces are discharged spontaneously, the stools consisting wholly of intestinal mucus mixed with more or less blood. The hepatic and cutaneous functions are invariably deranged in this affection, neither bile being seen in the evacuations nor perspiration on the skin at any time during the active period of the malady. For a long time scybala pent up in the colon were considered the principal cause of dysentery. A more enlightened pathology, however, has completely dissipated this error; it is now pretty well known that scybala are rarely present, and when present are not likely to produce the mischievous effects which have been heretofore so universally attributed to them. That this painful affection may be and is occasionally produced by obstructed perspiration from cold or vicissitudes of atmospheric temperature, I can easily imagine; but the principal and by far the most general agent I conceive to be malaria, and of this character are the cases I am now more especially considering.

To determine the true pathology of dysentery, we must look to the liver and to the mucous membrane of the intestinal canal; and here, as in the preceding diseases, we find the most unequivocal evidences of torpor and vascular engorgement. The worst cases are characterized by inflammation, and sometimes by ulceration.

Deducing my practice from what I conceive to be the true pathology of the disease, my primary object has been to correct as soon as possible the disordered state of the liver; and in carrying out this purpose, I have repeatedly had the satisfaction to find that the violence of the symptoms invariably subsided upon the restoration of the hepatic function. I give blue mass or calomel in combination with Dover's powder, at intervals of three, four or six hours. Auxiliary to this, I order a large emollient cataplasm, or, what I have sometimes thought preferable, a bladder half filled with warm water, to the abdomen. If the tenesmus be urgent, I direct anodyne enemata to be given. As soon as there is the slightest change in the dejections, or, to speak more definitely, when green or black bile begins to appear, I order a dose of castor oil, and repeat this every twenty-four or forty-eight hours, *pro re natâ.* I do not stop the mercurial treatment until I am satisfied that the healthy secretory function of the liver is established. In this disease we need have little fear of the specific effects of our medicine, as ptyalism is a rare occurrence. Of course, should this effect manifest itself, I would immediately suspend its further administration.

Dysentery often assumes a chronic form, and here a modified treatment becomes necessary. Counter-irritation, by means of blisters and tartar emetic frictions, must be had recourse to and perseveringly employed. At the same time considerable advantage will be derived from the use of the terebinthinates, with or without castor oil. Occasionally a little blue mass will be demanded to assist the liver, and to free the portal system from any remaining congestions. But in no form or stage of this disease can the soothing effects of opiates be dispensed with. For drinks and nourishment, the blandest articles are alone admissible; I prefer the preparations of gum, elm and arrow-root in the acute, and rice, thickened milk and mutton soup in the chronic forms of the disease. As convalescence is generally slow and protracted, I insist on my patients wearing flannel next the skin, and admonish them to guard by every means in their power against atmospheric changes.

I come now to speak of a class of diseases which, if not so dangerous as those already noticed, yet, constituting by their frequency more than a moiety of all with which we come in contact, are peculiarly worthy of consideration. That I refer to remitting and intermitting fevers it is almost superfluous to add. Although produced by the same causes and having many symptoms in common, the one disease often terminating in the other, and vice versa—being in fact essentially the same—yet, following the ordinary method of treating of these diseases, I shall notice them separately.

And first, of *Remitting Fever:*

This is generally ushered in with a chill, preceded for a longer or shorter period by diminution of appetite, slowness of the bowels and pains in the head and back. I have found this disease for the most part to occur under two modifications: the one characterized by a bitter taste in the mouth, tongue loaded with yellow mucus at first, but becoming dry and cracked in the progress of the fever, sense of weight and distress at the precordia, and frequent and copious vomitings of bile. The term *gastric* has been very appropriately applied to this form of the disease.

The other, or what is with equal propriety styled the *hepatic* form, is characterized by intense heat of the skin, violent pain in the head and early delirium, fullness and tension of the right hypochondrium, a clean tongue at first, great irritability of the stomach, frequent and forcible vomitings of a glairy-looking mucus *without any bile*, constipation of the bowels, yellowness of the skin and adnata, and the evacuation, sometimes spontaneously, but generally under the operation of medicine, of a black or tar-like matter from the bowels.

In this array of symptoms the discerning practitioner cannot fail to see the clearest evidences of venous engorgement and consequent functional disorder. That the biliary organs are most at fault will hardly admit of question. The fever is marked by exacerbations, two occurring for the most part in every twenty-four hours. The remissions generally take place morning and evening.

When called to a case of the gastric variety, if I find a good deal of nausea with imperfect emesis, I administer a dose of ipecac., and follow it up with plentiful draughts of warm water.

Pretty soon afterwards, and when the stomach is tranquil, I give a dose of calomel, combined or not, as the case may be, with rhubarb, and assist its operation if necessary with c. a. r. pills, Epsom salts or castor oil. In the generality of cases, however, the emetic may be dispensed with and the mercurial cathartic given at once. If the heat and thirst be great, I allow the patient cold water ad libitum, and not unfrequently add a little nitrate or supertartrate of potash to his drink.

Under this simple treatment I have oftentimes had the pleasure of seeing the fever not only mitigated, but its duration materially abridged. It is seldom necessary to continue active measures longer than three or four days. By this time, in a large majority of the cases, the disease will have yielded. For the remainder of the cure, an occasional laxative with proper regimen will generally suffice. If nervous symptoms at any time supervene, I do not hesitate to administer an opiate. Occasionally during a remission I have ventured on quinine, and although by no means certain that it has not proved beneficial, yet it is not my general practice to give it under these circumstances, nor, in truth, do I consider this or any other preparation of the bark at all essential to the cure of remitting fever. During convalescence it may answer very well in small doses as a tonic; but even here I prefer the chalybeates, the elixir vitriol, or cold infusions of chamomile or columba.

As respects the other or hepatic form of remitting fever, this depends so obviously on congestion and torpor, that I immediately set about correcting this state of things as fast as possible. With this view, I give mercurials, and follow them up with senna and salts, or some other pretty brisk cathartic. For the gastric irritability here, a dose of calomel is decidedly the best sedative. As the general heat is often excessive, I not only allow but encourage my patient in the free employment, both internally and externally, of cold water. As an application to the head, I frequently make use of ice. These remedies, it will be understood, are restricted to the period of exacerbation.

As there is evidently a greater amount of functional derangement in this than in the gastric variety already considered, it requires of course for its cure not only a longer but a more vigorous and persevering use of the means. A healthy restoration of the secretions may be regarded as a pretty sure index of convalescence.

Remitting fever may yield on the third, the fifth, or the seventh day from its accession, but in a great many cases it runs on to the ninth before a permanent improvement takes place. I am not in the habit generally of attaching much importance to what are called critical days, but I have so repeatedly seen this fever terminate as above, that I feel bound to subscribe to the truth of the observation, and to render my humble tribute to the Father of Medicine, whose superior acumen, more than two thousand years ago, enabled him to point it out. Dependent also on this same law of crises, is the fact of our patient's being better or worse on alternate days.

Particular symptoms in this as well as in almost every other disease require a modified treatment. A violent pain in the head or delirium, for instance, may make it expedient to apply a blister to the nape; or pain or great tenderness of the bowels may render the same application necessary to the abdomen. Epispastics, however, like quinine, I regard as constituting no part of the essential treatment of remitting fever. Should a cathartic at any time act too powerfully, or a tendency to diarrhea show itself, or the patient be unable to sleep, I invariably prescribe an opiate; if the bowels be constipated, however, opiates are inadmissible.

In the gastric variety of this disease, where the secretion of bile is superabundant and the vomiting excessive, I have not hesitated to give tincture of opium freely. It has appeared to me not only to allay the irritability of the stomach, but also to moderate the inordinate action of the liver. If nothing more than a truce is obtained by this practice, still I consider it justifiable, for having quieted the stomach, we are certainly better prepared to administer the remedies above detailed.

Now and then this disease assumes a continued or chronic form, with typhoid symptoms. This is manifested by a red and dry tongue, pain and tenderness of the bowels, diarrhea, delirium and subsultus. This I have found decidedly the most dangerous form under which remitting fever is presented to our notice. The diarrhea is especially troublesome. The treatment here consists principally in opiates and counter-irritants. As this form of the disease proves sometimes exceedingly obstinate, and often puts on many anomalous symptoms, we are driven to the necessity of making frequent changes in our prescriptions.

I have alluded to two forms or varieties under which remitting fever occurs, viz., the *gastric* and the *hepatic*. I cannot help thinking that a real pathological difference exists, and that a large majority of the cases may be recognised as belonging to either the one form or the other. It frequently happens that we are unable to perceive this difference, the distinctive character of each being destroyed, either by the commingling of symptoms, or by the obscurity or entire absence of the pathognomonic signs. There is no difference, however, so far as treatment is concerned; the same general indications are to be fulfilled in both cases, and hence the discriminating physician will be at no loss to see his way clearly, and to address his remedies accordingly.

I proceed next to the consideration of *Intermitting Fever:* While here, in New Albany, this occurs about as often as any other form of fever, in the *country* it is emphatically *the fever*. The quotidian and tertian are the types under which we generally meet with it. In some seasons, as they were in the last, for instance, double tertians are frequent. The symptoms which characterize the forming stage of an intermittent paroxysm do not differ from those which usually precede the development of other forms of fever. A sense of lassitude, frequent yawning and stretching, a feeling of uncomfortable weariness of the whole body, and slight aching pains in the loins and extremities, constitute the earliest manifestations of its approach.

The fit or paroxysm consists of three stages, a cold, a hot and a sweating stage; these generally succeed each other with remarkable regularity and in the order here enumerated.

Finding the gastric and hepatic symptoms giving character here as well as in the case of remittents, I am in the habit of directing my therapeutics during the paroxysm according to the predominance of the one or the other. During the chill or cold stage, I employ external warmth, and allow the patient hot drinks if the stomach will retain them. If there is much gastric distress and vomiting of bile, I apply a sinapism to the epigastrium and give some tincture of opium, and at the same time prohibit the use of drinks. This course I have found not only to allay the irritability of the stomach, but likewise, by anticipating the subsequent stages, materially to shorten the paroxysm.

When reaction is established, I give a dose of calomel and rhubarb, and follow it in a few hours if necessary with fresh-made R.A.C. pills. If the thirst be great, I permit the use of cold drinks; and if there be much febrile heat, with pain in the head, I order the free external use of cold water. Patients almost universally express their satisfaction with this remedy; it is exceedingly grateful to their feelings, and is generally what they most desire. Employed under the restrictions referred to, I have never seen it do harm, but, on the contrary, incalculable good. I allow acidulated drinks where the patient has a particular craving for them, but ordinarily I prefer toast-water or any of the common teas. When the hot is about to be succeeded by the sweating stage, I suspend the cold water treatment, and for the remainder of the paroxysm direct all the drinks and nourishment to be taken warm.

In the other condition alluded to, or when the cold stage is ushered in by continued but fruitless efforts to vomit, indicating most conclusively an aggravated state of internal congestion, I begin immediately with mercurial cathartics, using at the same time, as in the first case, sinapisms to the stomach and extremities, and external warmth. My object is to get the liver to acting as soon as possible. The evacuations in almost every instance will be found very dark. If these do not change to a more healthy appearance by the close of the paroxysm, I repeat the mercurial once or oftener during the apyrexia. Here, too, I make free use of cold water when the required conditions are present, and in general pursue a treatment in the hot and sweating stages similar to what has been already detailed.

If it be a first paroxysm, as we do not yet know what may be the particular type of the fever, I seldom resort to quinine. When, however, the type is ascertained, we can generally see our way clear enough. Having premised a mercurial cathartic or two, for the purpose of evacuating the bowels and relieving congestion, in a vast majority of cases the cure may be completed with quinine alone. Under the necessary qualifications and restrictions, therefore, this medicine may be regarded as a specific for intermittent fever. To make assurance doubly sure, however, I am in the habit of directing the patient to be well wrapped up in bed three or four hours before the expected chill—to commence with the quinine, and take, if an adult, two, three or four grains every hour until six, nine or twelve grains are taken, or until the recurrence of the chill makes it necessary to suspend the medicine.

In conjunction with these means, I order mustard plaster or horseradish to be bound to the extremities, and allow the patient the free use, if the stomach will retain them, of warm aromatic teas. If a perspiration is not readily excited, I give the patient a teaspoonful of the camphorated tincture of opium, or twenty-five drops of laudanum, and have warm bricks or bottles applied to his feet, and if necessary order an additional supply of bedclothes. This plan, if sedulously carried out, I believe will seldom fail.

Occasionally a case is met with that seems to defy the anti-periodic virtues of our remedy. The best we can do in these circumstances is to omit the quinine altogether, and to trust to the joint employment of calomel, Dover's powder and blisters. A slight ptyalism will sometimes cause the disease to yield after every approved remedy has failed.

Quinine so generally suceeeds in curing intermittents that some physicians think we have no other remedy. Admitting it to be the chief and the most important, it is not, however, the only one. Emetics, opium and stimulating cathartics, administered a short time before an expected paroxysm, have each repeatedly proved successful. Every season we hear of numbers who have been cured of the chills without a particle of quinine. After a mercurial cathartic, a single dose of Dover's powder timely administered will very often effect a cure; this I have repeatedly witnessed in my own practice.

Having arrested a paroxysm of intermittent fever, we should be careful not to suspend our remedial measures too soon. It is best to continue the quinine, interposing an occasional purgative, for several days during convalescence. In most cases this will be indispensably necessary to insure an immunity from a recurrence of the disease.

Thus much for our warm-weather diseases as they ordinarily present themselves to our notice. The task assigned myself, however, would be very imperfectly accomplished were I to omit to speak of a certain form the intermittent sometimes assumes, and which, in truth, is almost the only form of the disease that is really dangerous. The reader will of course understand me as having reference to what is popularly known as the *congestive chill.*

This is characterized mainly by a prolonged cold stage, ordinarily with little and sometimes without any febrile reaction. We have here, in their most pronounced as well as alarming aspects, all the evidences of the most perfect and overwhelming engorgement of the large vessels of the interior. The epithet "suffocated excitement," is most felicitously applied by Dr. Rush to this peculiar state. There is no mistaking it, for he who has seen it once will find no difficulty in recognising it for ever afterwards.

While the congestion lasts, the skin is cold and generally covered with profuse perspiration; the thirst is intense; the pulse is weak, and often scarcely perceptible; the countenance is either pale or of a modena color, and expressive of the most intense suffering. The patient refers almost all his sickness to an indescribable feeling about his heart; he heaves and sighs for breath, and calls for the doors and windows to be opened; he tosses his arms about and throws off the bed-clothes with violence; he is constantly turning himself in bed, but finds no relief in a change of posture; if not restrained, he will spring up and walk about the room, and do this sometimes even when pulseless. In addition to all this, he is frequently tormented with the most deadly sickness at the stomach, and makes repeated ineffectual efforts to vomit. In all my professional experience I have never had my sympathies aroused so much as when witnessing cases of this kind. The scene is emphatically a death-struggle, and which way victory will turn it is impossible to tell, such is the awful uncertainty of the issue.

Often the patient dies in the second paroxysm, seldom does he survive the third. Coming upon him suddenly, and as it were with the power of a giant, the disease seems to prostrate and paralyze all the energies of the individual, and to hold him completely captive.

After six, eight or twelve hours, or it may be even longer, a slight degree of warmth begins to manifest itself on the surface, and the pulsations at the wrist can be felt a little more distinctly. The patient breathes easier and complains less of the precordial distress. The stage of excitement has now set in, and with its development the signs of internal plethora partially and for the time being disappear. This stage may be of longer or shorter duration. The more full and perfect the reaction, the better it is for our patient.

The congestive chill may attend a quotidian, or a tertian, or a double tertian, as I have had frequent opportunities of witnessing during the past season; but let it occur when it will or in connection with what particular type it may, it is always to be regarded as a most dangerous accompaniment of fever.

As respects the treatment of this most formidable disease, I have but little to say. Quinine and calomel are the remedies in which I place the greatest confidence. As the disease is rapid in its work, the remedies must be correspondingly prompt and active. If called to a patient in the chill, I immediately give a large dose of quinine and laudanum, and employ sinapisms and external warmth most assiduously. The cold dash, so highly recommended by some, I have never tried, and should hesitate long, no doubt too long, before I would resort to it. Should considerable reaction follow the chill, however, attended with a dry and burning skin, as is sometimes the case, I have found sponging with cold water both safe and useful, and patients never object to its employment.

As soon as the patient is out of the paroxysm, I begin with quinine and calomel, and give four or five grains of each every two, three or four hours, continuing them until the apyrexial period is past, and sometimes for a day or two longer. If in the mean time ptyalism shows itself, I withdraw the mineral and continue the quinine alone for a longer or shorter period, according to the exigencies of the case. We should not fail to apply a large epispastic to the stomach five or six hours in advance of the anticipated chill, as by its stimulant and revulsive properties, it will aid materially in preventing a paroxysm. If these measures do not succeed, I question much whether any will—at least such has been my experience.

In the above detail of the remedies applicable to the treatment of our warm-weather diseases, it cannot have escaped observation that no mention whatever is made of venesection. The reason simply is, that I have ceased to regard the lancet as a necessary remedial measure. In former years I was much in the habit of bleeding patients in the hot stage of fevers, and especially in cases where considerable local pain was complained of; but for the last two seasons I have not resorted to this means in a single instance, and many of my patients, too, were precisely in the condition in which I once thought venesection indispensable. These having recovered as readily as the others, and with their strength certainly less impaired, I have come to the conclusion that bleeding is not necessary, and being unnecessary, I think it should no longer be regarded as a remedy for our miasmatic diseases. But that I may not be understood as unconditionally proscriptive, I would qualify this remark by observing, that should our summer diseases present themselves at any time with symptoms decidedly inflammatory, I should feel that I was not doing justice either to myself or my patient, were I to neglect to call to my aid so efficient an auxiliary as the lancet.

It has been asserted, in view of our locality, that New Albany is unhealthy. Before concluding, I would say a word on this point. Although it would not be difficult to show that the assertion has been made unadvisedly, yet even admitting it to be true that we have a considerable amount of sickness, still the fact is well known to all conversant with our diseases, that they are perfectly amenable to medical treatment. If the number of deaths per annum be any criterion of the health of a place, then has New Albany nothing to fear from a fair comparison with other cities. It is very certain that, in proportion to the population, the mortality with us is small.

What proportion of the deaths is due to the particular diseases discussed in this paper I have had no means of ascertaining. I have been enabled, however, by an inspection of the city sexton's register, to make out the following statistics. Embracing a term of five consecutive years, they exhibit pretty accurately the amount of mortality during this period within the corporation limits. As the census is taken only at long intervals, the precise population for each year could not be ascertained; I have been under the necessity, therefore, of assuming an average number for the whole period.

| Year. | Number of deaths per annum. | Population. Average for five years. | Deaths per cent. | Proportion of deaths to pop. for five years. |
|---|---|---|---|---|
| 1842 | 113 | 4,800 | $2\frac{1}{3}$ | |
| 1843 | 103 | 4,800 | $2\frac{1}{7}$ | |
| 1844 | 89 | 4,800 | $1\frac{7}{8}$ | as 1 for 47 |
| 1845 | 82 | 4,800 | $1\frac{7}{9}$ | |
| 1846 | 122 | 4.800 | $2\frac{1}{2}$ | |
| 5 years. | 509 | 24,000 | $2\frac{1}{8}$ | 47 |

Thus it appears that the number of deaths, per centum, for the last five years is $2\frac{1}{8}$, or one for every forty-seven inhabitants. When it is recollected that this estimate embraces the deaths from every cause, accident as well as disease, the per centage is certainly very moderate.

Having taken some pains to examine the bills of mortality for a number of localities reputed healthy, I find on comparison that we are above the general average, and consequently that on the score of salubrity New Albany deserves to be ranked among the most highly favored cities of the land.

